# Design of Elastomer-CNT Film Photoactuators for Nanolithography

**DOI:** 10.3390/polym11020314

**Published:** 2019-02-13

**Authors:** Le Li, Zhongjie Huang, YuHuang Wang, Keith A. Brown

**Affiliations:** 1 Department of Mechanical Engineering, Boston University, Boston, MA 02215, USA; leli1990@bu.edu; 2 Department of Chemistry and Biochemistry, University of Maryland, College Park, MD 20742, USA; huangz@umd.edu; 3 Division of Materials Science & Engineering and Physics Department, Boston University, Boston, MA 02215, USA

**Keywords:** photoactive thin-films, elastomer-nanotube composites, polymer pen lithography, nanocomposite, photoactuator, soft robotics

## Abstract

Polymer pen lithography (PPL) is an approach to multiplexing scanning probe lithography, in which an array of probes on a compliant film-coated rigid substrate are used to write patterns on a surface. Recently, it was shown that these nominally passive pen arrays can be rendered photo-active by making them out of a polydimethylsiloxane (PDMS)–carbon nanotube (CNT) composite. While such photoactuated pens in principle represent a rapid, maskless, and versatile nanomanufacturing strategy, a key challenge that remains is learning how to effectively control the writing of each pen, individually. In this research, we studied the design of PDMS–CNT thin-film photoactuators and experimentally explored the role of illumination radius, film thickness, and CNT concentration. Additionally, we have proposed a model that predicts actuation efficiency, actuation time, and the crosstalk between pens. Based upon these results, we have generated a map of working efficiency to elucidate the ideal choice for specific actuation requirements. This work lays the foundation for studying further photoactuatable composite films as actuators in applications beyond lithography including soft robotics and adaptive optics.

## 1. Introduction

Breakthroughs in nanotechnology have continued over the past decades, partly due to the development of advanced forms of nanolithography, such as scanning probe lithography, nanoimprint lithography, and electron beam lithography [1,2,3,4,5,6]. While these techniques are able to pattern at high-resolution, they have drawbacks, either in terms of requiring a mask to define a pattern, requiring costly equipment, having limited throughput, or needing many complex processing steps. As an example, dip-pen nanolithography (DPN) was first introduced as a maskless nanolithography technique that uses the tip of a commercially available scanning probe to physically transport different kinds of inks (molecular inks, hydrogels, lipids, etc.) onto a variety of substrates [7,8,9,10]. While this technique allows direct writing in a simple process, the low throughput inherent to serial writing remains a central barrier. In order to overcome this challenge, polymer pen lithography (PPL) was introduced as a high throughput scanning probe technique that integrates millions of elastomeric pens on a rigid backing layer, coated with a compliant film that serves the role of the cantilever in DPN [11,12,13,14,15,16,17,18]. While this technique dramatically improves upon the throughput of single-pen DPN through parallelization, conventional PPL utilizes an array of passive pens that cannot define substantially different features than their neighbors, thus limiting the generality of the approach [18]. Thus, a major challenge barring the use of PPL as a general nanopatterning tool is the development of scalable approaches to actuate individual pens relative to their neighbors, within an array.

**Figure 1:**
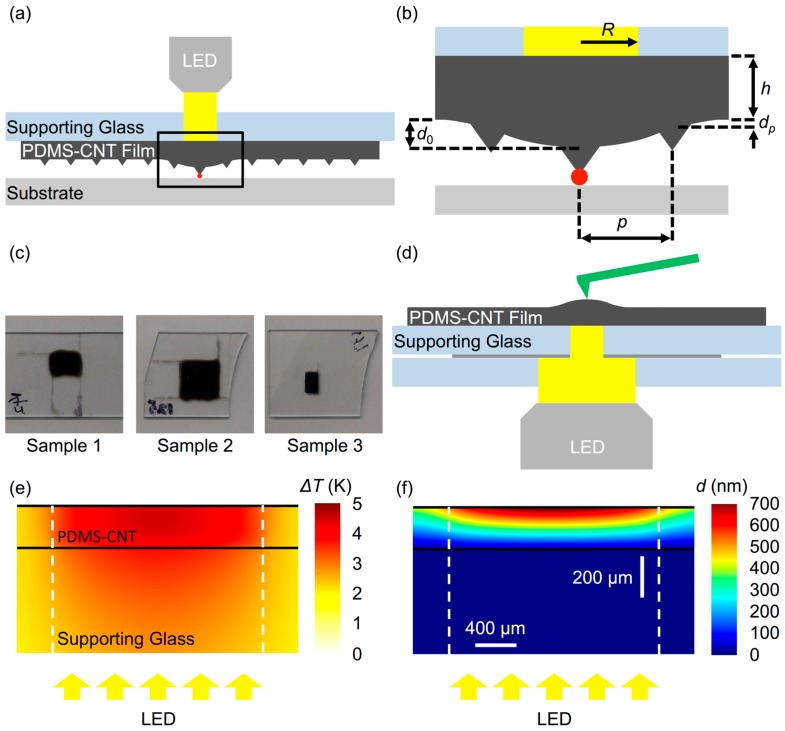
Schematics of (**a**) working principle of polydimethylsiloxane-carbon nanotube (PDMS–CNT)-based photoactuated polymer pen lithography (PPL). When illuminated, the desired pen deformed out-of-plane, to come into contact with the substrate and transfer ink. (**b**) Critical geometric factors of a photoactuated PPL pen array, including illumination radius R, elastomer film thickness h, and pen-to-pen distance p Crosstalk occurred when the pen that neighbors the illuminated pen deformed, potentially giving rise to unintended tip-sample contact. (**c**) Photographs of PDMS–CNT films under study: 0.25 CNT wt %, *h* = 204 μm for Sample 1; 0.5 CNT wt %, *h* = 99 μm for Sample 2; 1 CNT wt %, *h* = 37 μm for Sample 3. (**d**) Schematic of experimental setup where an atomic force microscope (AFM) probe was positioned on top of the PDMS–CNT film that was illuminated from beneath, using a broadband LED light source. Typical (**e**) temperature Δ*T* map and (**f**) vertical deformation d map of a system with h=  200 μm, *C*_CNT_ = 0.25%, R= 1 mm and illumination intensity $I_{0}=$ 0.45 W/cm^2^. Scale bars apply to both (**e**,**f**).

Acknowledging the importance of the actuation problem, prior work has focused on realizing single pen actuation through actuation systems built into the pen array, including thermal actuators that expand the elastomeric backing layer using Joule heating and pneumatic channels that inflate when pressure is applied [19,20]. While both of these techniques provide an accurate control over the position of pens, the challenge of making independent electrical or pneumatic connections to each pen limits the scalability of these strategies when considering arrays of thousands or even millions of pens. Instead of integrating complex physical systems into the pen array, utilizing a photoresponsive material to affect actuation drastically simplifies the structure of the pen array as it can be simple with an external projection system, such as a digital micromirror device, selecting active pens in a scalable fashion [21]. In previous work, pen arrays composed of a polydimethylsiloxane (PDMS)–carbon nanotube (CNT) composite, which has been widely studied as a photomechanical material [22,23], have been successfully used to print ink in a photoactuated fashion [24]. An actuation efficiency of 200 nm/mW at a time scale of <1 s was observed when illuminating a 204 μm thick PDMS–CNT pen array with a 120 μm radius circular beam of light (Figure 1a). The mechanism of this phenomenon was investigated through a combined experimental and computational method, and was found to be consistent with the conversion of the optical power absorbed by CNTs into heat, which thermally expanded the elastomer. While, with this photoactuation, this composite was powerful and fast enough to print, it lacked the selectivity to actuate individual pens.

In this article, we have presented a combined numerical and experimental approach for optimizing PDMS–CNT thin-film photoactuators and have determined the criteria for single pen photoactuation. The overarching motivation for this work was to realize photoactuated lithography, using polymer pens, which requires that the response time should be <1 s and to be commensurate with other steps in the writing process, and that the pen deformation ($d_{0}$) should be at least one micrometer larger than that of the neighboring pen ($d_{p}$), that is, $d_{0}-d_{p}$ > 1 μm, to avoid crosstalk. To determine the conditions that satisfied these requirements, we began with a series of atomic force microscope (AFM)-based experiments on the PDMS–CNT films to measure the out-of-plane deformation in response to illumination and determined the magnitude of actuation and crosstalk. Subsequently, we performed comprehensive finite element analysis (FEA) solving both heat transfer and thermal expansion and found agreement with experiments in terms of actuation efficiency, actuation time, and crosstalk. Using this experimentally validated model, we predicted the optimum CNT concentration, film thickness, and illumination radius to mitigate crosstalk and realize individual pen actuation, in a general sense. Ultimately, the goal of this work is to obtain a set of design guidelines that will aid in the development of thin-film photoactuators for lithography, soft robotics, and adaptive optics [25].

## 2. Materials and Methods

### 2.1. Fabrication of PDMS–CNT Composite Films

Multiwall CNTs (SMW 100, SouthWest NanoTechnologies, Norman, OK, USA) were initially functionalized with –(CH_2_)_5_CH_3_ groups via five cycles of Billups–Birch alkylation [26] to enhance their solubility in chloroform. This process was then followed by dissolving both the treated CNTs and the PDMS base (Sylgard 184, Dow Corning, Midland, MI, USA) in chloroform and stirring to thoroughly mix them at a specified CNT weight fraction *C*_CNT_. The solvent was gradually removed by magnetic stirring for 72 h in an uncapped vial. PDMS crosslinker (5:1 *w*/*w* base to crosslinker) was then added into the PDMS–CNT mixture. Bubbles within the mixture were removed under vacuum before drop-casting the mixture onto glass slides that had been pre-cleaned in 1% aqueous solution of Alconox, nanopure water, ethanol, and acetone, sequentially. The PDMS–CNT composites were then cured in an oven at 80 °C, for 4 h. Following curing, the film height *h* was determined using a stylus profiler and white light interferometry microscopy. The three film samples investigated in this paper were made of different CNT weight fractions (Figure 1c), 0.25 wt % for Sample 1 (h=204±5.5 μm), 0.5 wt % for Sample 2 (h=99.1±2.3 μm), and 1 wt % for Sample 3 (h=36.8±2.6 μm); all absorbing more than 95% of the light at 578 nm.

### 2.2. AFM Characterization

A customized AFM system (MFP-3D Infinity, Asylum Research, Santa Barbara, CA, USA) was employed to study the photomechanics of the PDMS–CNT composite films (Figure 1d). Specifically, a broadband LED source (MCWHF2, Thorlabs, Newton, NJ, USA) was incorporated into the base of the AFM to locally illuminate the composite films with a light intensity $I_{0}=0.45$ W/cm^2^. In order to localize the illumination region, glass slides were prepared with aluminum films that included transparent holes, which were made using photolithography, to specify an illumination radius *R* to be either 1 mm, 500 μm, 250 μm, or 120 μm (Figure 1d). The probe was scanned in tapping mode in a 10×10 nm^2^ area, such that the tip was effectively static on the substrate while the feedback tracked the motion of the surface in the vertical direction [19,24,27]. The scan rate of the AFM probe was 0.1 Hz to capture the dynamics of deformation while the AFM executed a single scan line. The incident light was periodically cycled by controlling the LED source using a function generator that was also recorded by the AFM system to synchronize the film deformation with illumination.

### 2.3. Finite Element Analysis

FEA was performed using commercial software (COMSOL 5.3a, COMSOL Inc., Stockholm, Sweden). Specifically, a 2D axisymmetric model was created in which a PDMS film with a central heating region was positioned on top of a glass slide. A volumetric heating source in the center region of the PDMS was employed, which exponentially decayed along the axial direction to simulate light absorption by CNTs based on the Beer–Lambert law where transmitted light intensity follows $I\left(z\right)\;=I_{0}exp\left(-\beta C_{\mathrm{CNT}}z\right)$. Here, $I_{0}$ is the incident light intensity and β is a CNT-concentration-dependent light absorption coefficient, which was found to be β= 10 (μm·%)^−1^, by measuring the total light absorption through PDMS–CNT films [28]. A fully constrained boundary condition was applied to the film–glass interface. Mechanical properties of the PDMS, including the thermal conductivity and thermal expansion coefficient, were set to the tabulated values of pure PDMS as the CNT concentrations in the PDMS were too low to substantively change these properties [29]. The implication is that, in this model, CNTs only influence the system by absorbing light and not by otherwise affecting the properties of the films. FEA results in a map of temperature (Figure 1e) and vertical deformation (Figure 1f). 

## 3. Results and discussion

**Figure 2:**
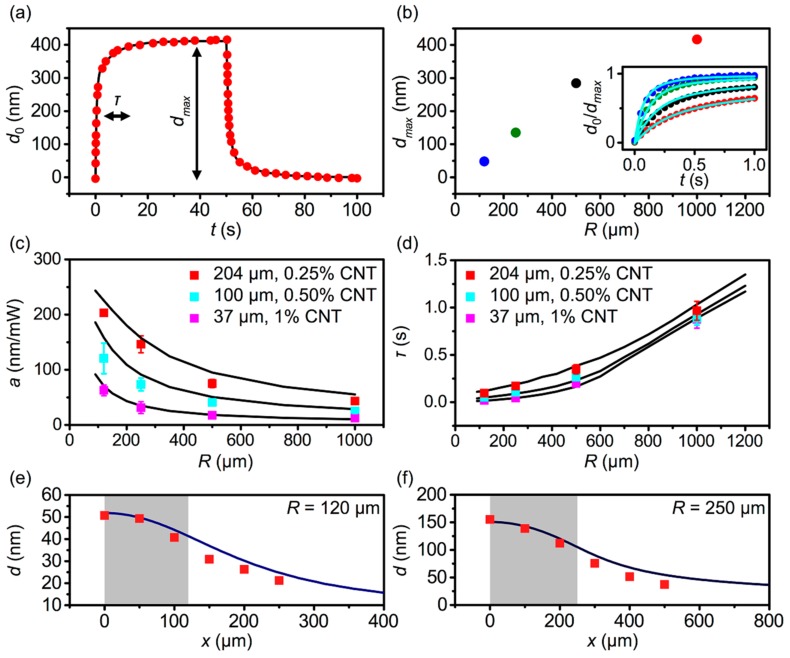
Characterization of photoactuation of PDMS–CNT films, based upon a combined experimental and computational method. (**a**) Typical trajectory of a h=204 μm PDMS–CNT composite film, under an illumination intensity $I_{0}=0.45$ W/cm^2^ with R=1 mm. At a time t=50 s, after the onset of illumination, the deformation is denoted by $d_{max}$, with a characteristic rise time τ. (**b**) $d_{max}$ obtained from the same film under a variety of R ranging from 120 μm (blue), 250 μm (green), 500 μm (black), to 1 mm (red). The inset shows $d_{0}/d_{max}$ trajectories for these experiments. (**c**) Actuation efficiency ($a=d_{max}/P$, where P is the incident optical power) and (**d**) τ *vs.* R. Profiles of *d* vs. position *x* of h=204 μm PDMS–CNT with (**e**) R=120 μm and (**f**) R=250 μm. Grey regions represent the illuminated areas. In all panels, the dots represent results from experiments and solid lines indicate results from the finite element analysis (FEA).

In order to begin exploring the conditions for efficient photoactuation with minimal crosstalk, we hypothesized that the size of the illumination area, as parameterized by *R*, plays a critical role. In a typical AFM experiment to determine the out-of-plane deformation of PDMS–CNT films caused by illumination, the motion of the AFM probe, when light was on, revealed a clear photoactuation (Figure 2a). Specifically, $d_{0}>$ 400 nm in 50 s was observed with $I_{0}=$ 0.45 W/cm^2^ and R=1 mm. In order to realize individual pen actuation, R should be less than the pen-to-pen distance (p), which is usually in a range 100 to 1000 μm, thus, it was important to study actuation with a smaller *R*. While keeping $I_{0}$ constant, we varied R by placing aluminum pinholes beneath the PDMS–CNT film. With R=0.12 mm, $d_{max}$, which represents the deformation $d_{0}$ at t= 50 s, decreased drastically to 50 nm, which was reflective of the smaller total power delivered through the reduced area of illumination. Surprisingly, when defining actuation efficiency, $a=d_{max}/P$ where P is the total optical power, an opposite trend was observed, as a increased from a=43 nm/mW to 200 nm/mW. This indicated that a smaller R led to a substantially more efficient actuation. Additionally, defining a time constant τ to describe the delay after the onset of illumination at which the film deformed by $0.64d_{max}$, a faster response was observed for a smaller R, specifically, τ decreased from 1 s to 90 ms as R decreased from 1 mm to 120 μm.

In addition to details of the illumination, the composition and geometry of the PDMS–CNT composite film are expected to play a major role in determining the effectiveness of a photoactuator. In order to systematically study these factors, we performed a series of experiments on actuators with different *h* and *C*_CNT_. By testing three samples with varying *h* and *C*_CNT_ (Figure 2c,d), we confirmed that a increases and τ decreases with decreasing R. Perhaps more interestingly, thinner films always experienced a smaller d but also a smaller τ under the same experimental conditions, indicating that they were less efficient, but faster. Specifically, a decreased to 120 nm/mW for sample 2 (h=100 um) and 60 nm/mW for sample 3 (h=37 um), when R=120 μm, however, τ became 49 ms and 20 ms, under the same illumination conditions. These results suggest that thicker films might be desirable so long as their actuation speed is sufficiently rapid. 

While experiments represent an effective way to measure actuation, they are poorly suited as a tool to systematically optimize performance as sample preparation is a serial process. If, however, the experimental results could be recapitulated using FEA, then the FEA model could be used to optimize over a broad range of parameters. In order to determine whether experimentally observed actuation effects were consistent with the FEA simulations of thermal expansion, a series of FEA studies, based on a coupled heat transfer and thermal expansion model were performed using a model with the measured geometry, illumination conditions, and tabulated material properties. Specifically, time dependent models were constructed with (a) h=204 μm, $C_{\mathrm{CNT}}=$ 0.25%; (b) h=100 μm, $C_{\mathrm{CNT}}=\;0.5\%$; and (c) h=37 μm, $C_{\mathrm{CNT}}=1\%$, and tested in the range 100 μm <R< 1 mm. In order to model the heat generated from absorption of light by CNTs, an initial power density $I_{0}$ = 0.45 W/cm^2^ was set to match the experimental value, and a volumetric heating source was constructed, using an exponential distribution to reflect the Beer–Lambert type absorption. Good agreement was found between the computational results (solid lines in Figure 2b–d) and experimental data, in both a and τ, which confirmed that thermal expansion was the dominant effect and that the FEA model was able to predict the photoactuation of the PDMS–CNT composite films.

While the speed and magnitude of actuation is important, realizing independent actuation requires actuating a pen, relative to the neighboring pens. The motion of a neighboring pen is known as crosstalk and can limit the degree to which arbitrary patterns can be realized. In order to explore the magnitude of crosstalk, the deformation of a film (Sample 1) was experimentally measured at the center position of illumination and at positions x that were 50, 100, 150, 200, and 250 μm off center, as determined by a linear translation stage (Figure 2e). The illumination region (R = 120 μm) was centered at x = 0; denoted with a grey box. While it was clear that d decreased as x increased, even at x=250 μm, where the probe measured deformation 130 μm away from the edge of the illuminated region, d was found to be 20 nm, which indicated that substantial heat had diffused into this region of the film and caused a deformation. The predicted surface profile from the FEA was also found to exhibit this same trend and was in quantitative agreement with experiment. The magnitude of crosstalk was substantially larger when a larger illumination area was used (R=250 μm, Figure 2f).

**Figure 3:**
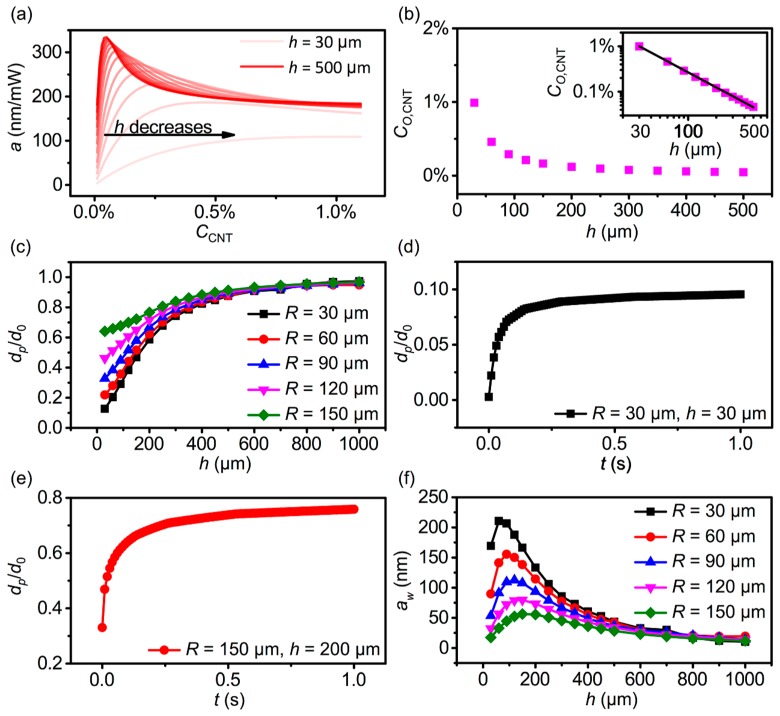
Optimization of PDMS–CNT films on the basis of FEA. (**a**) Actuation efficiency a vs. $C_{\mathrm{CNT}}$ for various h with R = 60 μm. (**b**) Optimal CNT concentration $C_{O,\mathrm{CNT}}$ vs. h and (inset) the power law relationship between these quantities. (**c**) Crosstalk magnitude $d_{p}/d_{0}$ where p=150 μm plotted vs. h with various values of R. Time dependency of $d_{p}/d_{0}$ for systems with (**d**) R = 30 μm and h = 30 μm compared with (**e**) R = 150 μm and h = 200 μm. (**f**) Calculated working efficiency $a_{w}$ $=\left(d_{0}-d_{p}\right)/P$ vs. *h*.

Given confidence that this computational model allowed us to understand the mechanism underlying PDMS–CNT composite film photoactuation and predict its actuation efficiency, time constant, and crosstalk, we hypothesized that it could be used to optimize photoactuator performance to approach individual pen actuation. As an initial study, we sought to optimize *C*_CNT_ by performing a series of FEA calculations, with varied $C_{\mathrm{CNT}}$ and h, under constant R= 60 μm (Figure 3a). These calculations were performed using the experimentally determined β. Interestingly, these calculations suggested that as h changed, the largest a occurred at different $C_{\mathrm{CNT}}$, which we denoted as $C_{O,\mathrm{CNT}}$. When plotting $C_{O,\mathrm{CNT}}$ *vs.* h (Figure 3b) the data were found to be well-described by the power law fit $C_{O,\mathrm{CNT}}\left(\%\right)={\left(h/30\;\mu m\right)}^{-1.1}$. Interestingly, this finding along with the Beer–Lambert relationship, indicated that efficiency was maximized when ~96% of the light was absorbed. To rationalize this result, one can consider that an ideal photothermal actuator would deliver heat uniformly throughout the thickness of the film in the illuminated region. If the CNT concentration was too large, light absorption would be highly localized near the glass slide and the resultant heat would be preferentially drawn out through the glass. If, in contrast, the CNT concentration was too small, then the total absorption would be too low and a large fraction of the light would pass through the film without being absorbed. Thus, the 96% attenuation result represented a balance between attempting to distribute the heat generation evenly in the film and capturing most of the incident light.

In addition to optimizing photoacuator efficiency, we performed FEA to identify conditions that minimized the crosstalk. In order to identify conditions with a minimal crosstalk but high efficiency, a series of FEA calculations were performed with R ranging from 30 to 150 μm, and h from 30 μm to 1 mm, using previously identified $C_{O,\mathrm{CNT}}$ for each *h*. Taking p= 150 μm to be a typical value for a PPL pen array, we studied $d_{p}/d_{0}$ *vs.* h, with a varying R (Figure 3c). Interestingly, $d_{p}/d_{0}$ increased with increasing R and h, suggesting that minimizing the size of the system, relative to *p*, would reduce crosstalk. The dependence on size suggested that the dynamics of different-sized systems could be important because of the dependence on thermal diffusion. Thus, the dynamics of $d_{p}/d_{0}$ were also studied when R= 30 μm and h = 30 μm (Figure 3d), and when R= 150 μm and h = 200 μm (Figure 3e). These results indicated that a longer time was required for heat to equilibrate in larger systems, even with *p* being held constant. While crosstalk occurred faster for the smaller system, $d_{p}/d_{0}$ saturated at a value of 0.11, for the smaller system, compared to 0.76 for the larger system. Thus, crosstalk was substantially worse in larger systems, suggesting that using extremely short illumination times in a larger system would not be an effective path to limiting crosstalk.

While actuation magnitude and efficiency could be discussed separately in order to understand their physical origins, from a functional perspective, the important parameter that defined the operation of a pen array—how much can a given pen be actuated relative to its neighbors—is dependent upon both of these factors. Thus, we defined working efficiency $a_{w}$ = $\left(d_{0}-d_{p}\right)/P,$ which represents this composite property. Interestingly, when considering this parameter, there exists an optimum *h* for a given *R*, as thinner films are less efficient, while thicker films have more crosstalk (Figure 3f).

**Figure 4:**
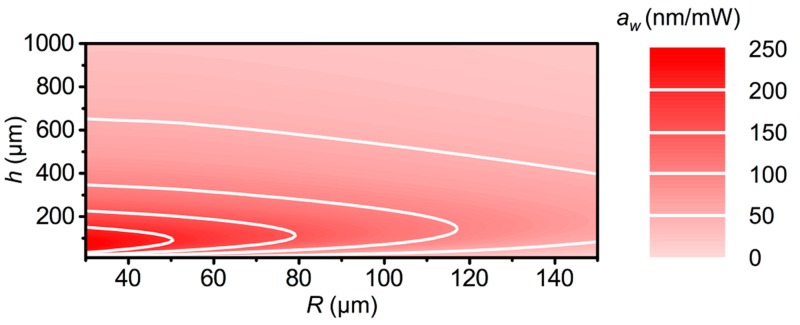
Map of $a_{w}$ vs. *h* and *R* with *p* = 150 μm. In all cases, $C_{O,\mathrm{CNT}}$ was chosen to be the optimized value.

More generally, this analysis allows one to produce a map of $a_{w}$ to represent all actuators with p=150 μm, which could potentially provide insight for using PDMS–CNT composite films as photoactuators (Figure 4). For example, to realize individual pen writing with a condition that $d_{0}-d_{p}=1$ μm, an h=100 μm PDMS–CNT-film, illumination with R=30 μm and P=4 mW was predicted to be sufficient. This analysis highlighted an important virtue of photoactuators in that they were more efficient than pure thermal actuation. Previously introduced thermal actuators, based upon Joule heating at the glass–PDMS interface, exhibited a maximum a= 100 nm/mW, which indicated that optimized photoactuators are ~2.5 times more effective, in addition to having a far simpler structure [27].

## 4. Conclusions

While PPL is a high-throughput technique that provides rapid and direct writing, patterning arbitrary features using this technique remains a key challenge. Photoactuatable composites have demonstrated the potential for addressing this challenge, by actuating pens using a light projection system. Here, we performed a series of experiments and computations to understand the role of geometry, illumination radius, and CNT concentration in determining the photoactuation performance of PDMS–CNT composite films. Our studies showed that crosstalk is related to the diffusion of heat from the illuminated region into non-illuminated regions. We predicted the optimum CNT concentration based on a balance between even heating of the film and capturing the majority of the incident light. Furthermore, we have provided a set of guidelines for choosing an optimum system for obtaining large actuation magnitudes with minimal crosstalk. Building on our prior demonstration that this approach can be used to define patterns in a photoactuated manner [24], the innovations and design rules presented here might lead to probes that can be actuated in a single probe manner. To parameterize this, we defined a working efficiency that described how much a given pen can be actuated, relative to its neighbors, per unit power. While we have focused on out-of-plane deformation as an enabler of scanning probe lithography, the results described herein are relevant to understanding any photothermal deformation of a thin film. Thus, this research work paves the way for the design of other photoactuatable systems such as styrene–isoprene block copolymers (SIS)–CNT and PDMS-carbon black [30]. A deeper understanding of these actuators has implications beyond lithography in fields including soft robotics, adaptive optics, and controlling vibrations [31,32,33,34].
